# An achiral ferromagnetic/chiral antiferromagnetic bilayer system leading to controllable size and density of skyrmions

**DOI:** 10.1038/s41598-019-39675-4

**Published:** 2019-02-27

**Authors:** F. J. Morvan, H. B. Luo, H. X. Yang, X. Zhang, Y. Zhou, G. P. Zhao, W. X. Xia, J. P. Liu

**Affiliations:** 1 CAS Key Laboratory of Magnetic Materials and Devices, Ningbo Institute of Material Technology and Engineering, Chinese Academy of Sciences, Ningbo, 315201 China; 2 Zhejiang Province Key Laboratory of Magnetic Materials and Application Technology, Ningbo Institute of Material Technology and Engineering, Chinese Academy of Sciences, Ningbo, 315201 China; 3 University of Chinese Academy of Sciences, 19A Yuquan Rd, Shijingshan District, Beijing, 100049 China; 4 School of Science and Engineering, The Chinese University of Hong Kong, Shenzhen, Guangdong 518172 China; 5 College of Physics and Electronic Engineering, Sichuan Normal University, Chengdu, 610068 China; 6 Department of Physics, University of Texas at Arlington, Arlington, TX 76019 USA

## Abstract

Magnetic skyrmions are topologically protected domain structures related to the Dzyaloshinskii-Moriya interaction (DMI). To understand how magnetic skyrmions occur under different circumstances, we propose a model for skyrmion formation in a bilayer system of ferromagnetic/antiferromagnetic (FM/AFM) films, in which the bulk DMI is only present in the AFM film. Micromagnetic simulations reveal that skyrmions are formed in this system due to the competition between the DMI and demagnetization energies. A critical interfacial exchange energy (*A*_*i*_ = 6.5 mJ/m^2^) is determined, above which the competition occurs at its full extent. More skyrmions are formed with increasing external magnetic field till a critical value above which the external field is too large and thus leading to the annihilation of skyrmions. The spacing between two skyrmions can be as small as 45 nm. Our results may give technological implications for future skyrmion applications.

## Introduction

Skyrmions have attracted great attention for both fundamental research and technological applications. Magnetic skyrmions exist in non-centrosymmetric bulk ferromagnets and magnetic thin films with a lack or breakdown of inversion symmetry^1^. Since their first experimental observation in 2009^2^, magnetic skyrmions have been found in bulk materials, including ferromagnets^2–7^, multiferroics^8^, and antiferromagnets^9–13^, as well as in ultrathin films^14^ and multilayers^15,16^. Magnetic skyrmions are widely investigated to understand their unique properties including the size, stability and the extremely low depinning current needed to move them^17–20^, namely 10^6^ A/m^2^ as compared to 10^12^ A/m^2^ for domain walls^1^. These properties make magnetic skyrmions very promising for the design of future spintronic devices, in which they can act as information carriers in memory and logic circuits^21–23^.

The stabilization of skyrmions mainly relies on the Dzyaloshinskii-Moriya interaction (DMI)^24,25^. Contrary to the Heisenberg exchange interaction which results in parallel or antiparallel alignment of magnetic moments [related to the dot product in the equation ***H*** = *J*_*ij*_∙(**S**_*i*_∙**S**_*j*_) with *J*_*ij*_ the exchange constant and **S** the spin moment], the DMI is an exchange interaction which favors magnetic moments perpendicular to each other [related to the cross product in the equation ***H***_DM_ = −**D**_*ij*_∙(**S**_*i*_ × **S**_*j*_) with **D**_*ij*_ the continuous effective DMI vector]. This characteristic is the reason why DMI is the key parameter for skyrmion stabilization: the topological protection comes from chirality, and DMI introduces chirality as its lowest energy favors spiral spin distribution, which can stabilize the skyrmion state^26^.

The DMI comes from an interplay of the spin-orbit coupling with the breaking of inversion symmetry^25^. A bilayer including a deposited magnetic thin film (usually Fe or Co) inherently has an acentric structure due to the fact that different materials are separated by the interface. If one of the film layers is chosen to be heavy metals with strong spin-orbit coupling, large DMI may occur between magnetic atoms adjacent to the interface due to spin-orbit scattering according to the Fert-Levy model^27,28^. The strong ferromagnetic interaction in these films is beneficial to room-temperature applications. However, the DMI is basically confined in one or two atomic layers beside the interface. To produce and stabilize skyrmions, the film should be ultrathin and the interface should be flat enough. There should be very limited atomic diffusion and defects in the system^29,30^, so that the DMI level can be kept to stabilize the skyrmions. In comparison, many bulk magnetic materials with acentric structures, such as MnSi^2^, FeGe^31^, Cu_2_OSeO_3_^8^, and GaV_4_S_8_^32^, have been found having DMI. These materials do not contain heavy elements, so that spin-orbit coupling, and therefore DMI, are not as strong as those in the aforementioned case. Nonetheless, unlike interfacial DMI, bulk DMI prevails in the crystals, which is not limited by the space distribution of DMI. However, it needs to be emphasized that most of these materials have a low Curie temperature and low anisotropy due to weak FM interactions. Researchers even turned to some materials without DMI, where skyrmions or biskyrmions were stabilized well beyond room temperature by magnetostatic interaction^15,33^, albeit their geometry dependence is not easy to control.

Understanding these different mechanisms of skyrmion formation is beneficial for the manipulation of skyrmions in a controllable system. We made attempts to establish such a system. To start, we note that DMI is originally found in magnetic oxides with strong antiferromagnetic (AFM) interactions^24^, which comes from the fact that many oxides have complex structures with a low symmetry. For instance, the multiferroic BiFeO_3_ has long been found with strong DMI^34–37^. Neutron scattering measurements showed that it exhibits a *G*-type AFM structure modulated by a Néel-type spin spiral^34,35^. Indeed, the possibility to manipulate skyrmions in *G*-type AFM structures through spin transfer torques^10,11^ as well as the thermal stability of skyrmions in *G*-type AFM structures have been investigated^10^, where the Magnus force was surely cancelled. Actually, the creation of an AFM skyrmion by an external field should be an experimental challenge because the net magnetization of AFM materials equals zero. Note that AFM skyrmions cannot be generated without an effective field such as perpendicular magnetic anisotropy (PMA)^11^. However, if we introduce a model composite of an AFM matrix with an FM film on top of it, the effect of DMI may be transferred from the matrix to the FM film when a strong enough exchange coupling is present at the interface. The resulting chiral rotation in the FM film provides the chirality needed to stabilize skyrmions. As AFM materials with DMI are seldom explored to generate skyrmions, our aim is to make a wider use of this category of materials in skyrmionics. Besides, no heavy metal is needed to generate a locally high DMI in the FM film. The strong AFM interaction due to the superexchange in the matrix can also guarantee a high Néel temperature^38^. These appealing features make this new model promising for future experimental design.

In this paper, we present our model containing an achiral FM thin film on top of a chiral AFM film with bulk DMI. Micromagnetic simulations are used to analyze the influences of the interfacial exchange constant *A*_*i*_, DMI constant *D* in AFM film, *M*_S_ in FM film and an external field *H* on the magnetic structure. It should be emphasized that our model does not focus on any material in particular, giving us the opportunity to vary different magnetic parameters. The phase diagrams demonstrate that skyrmions can occur in a well-defined range of *M*_S_ and *D* through a weak interfacial exchange coupling *A*_*i*_. It is found that the competition between the FM film demagnetization energy and the AFM film DMI energy will naturally lead to the formation of a skyrmion cluster, while neither the AFM film nor the FM film is sufficient to stabilize a skyrmion state alone. Surprisingly, the number of generated skyrmions can be tuned by an external magnetic field, which is very important for future applications in skyrmionics.

## Results and Discussion

### Independent behavior of the FM and AFM films

**Figure 1:**
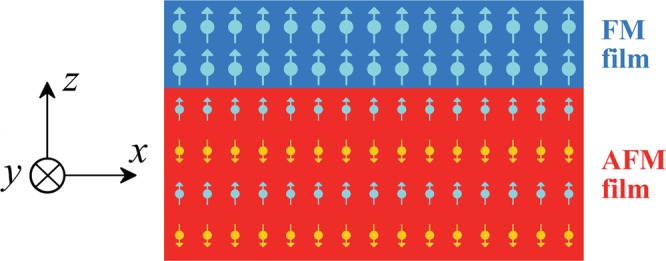
Schematic model of the ferromagnetic/antiferromagnetic system, with two and four atomic layers for the ferromagnetic and antiferromagnetic films, respectively. Our model has a square base with a 200-nm width, and a 3.36-nm height which represents six atomic layers. The magnetic moment distribution in the schematic corresponds to the initial state before relaxation.

**Figure 2:**
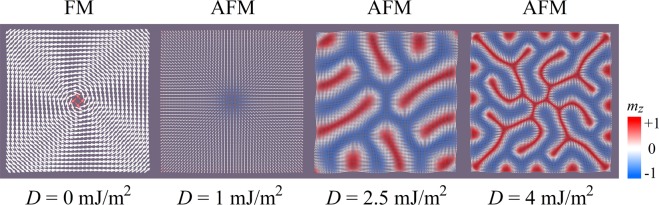
Magnetic moment distributions of the FM and AFM interface layers with *A*_*i*_ = 0 J/m^2^, for different *D* values. The color scale represents the out-of-plane component of the magnetization *m*_*z*_, which is used throughout this paper.

The proposed model, an FM/AFM system, along with the initial state used for the simulations, are schematically shown in Fig. 1. All the simulation details can be found in the Methods part. When discussing any energy, it is implied that this is the full model energy. Before simulating the exchange-coupled FM/AFM system, it is necessary to know how the magnetic moments behave independently in the FM and AFM films. To do that, we simply set the interfacial exchange constant *A*_*i*_ as zero, regarding that the top FM film has a very small magnetostatic influence on the AFM film. The results are shown in Fig. 2, and demonstrate that an FM film on its own only forms a vortex due to the reduction of magnetostatic energy. The vortex exists throughout the considered range of *M*_S_. The AFM film gives rise to Néel-type spirals induced by the DMI with the cycloidal plane vertical to the film plane, however no spiral appears for *D* = 1 mJ/m^2^ because *D* is low and the helix length is too large for the model to capture. The helix length *L*_D_ = 4π*A*/|*D*| was effectively found to be inversely proportional to the DMI constant according to our simulations^26^. From the patterns of the AFM film shown in Fig. 2 for *D* = 2.5 and 4 mJ/m^2^, the spiral periods are found to be 50 nm and 31 nm, respectively, which exactly matches the DMI helix length of 50.3 nm and 31.4 nm in our case.

The numerical results of the DMI energy are *E*_DMI_ = −2.6 × 10^−17^ and −6.7 × 10^−17^ J for *D* = 2.5 and 4 mJ/m^2^, respectively, for the AFM film. To compare them with the analytical results, we approximately model the patterns in the AFM film with ideal spirals, with the propagation vector along the *x* axis. The *M*_*x*_ and *M*_*z*_ components read: ${M}_{x}={M}_{{\rm{S}}}\,\sin \,\frac{-2\pi x}{{L}_{{\rm{D}}}}$ and ${M}_{z}={M}_{{\rm{S}}}\,\cos \,\frac{-2\pi x}{{L}_{{\rm{D}}}}$. By taking the DMI energy density, ${\omega }_{{\rm{D}}{\rm{M}}}=\frac{D}{{M}_{{\rm{S}}}^{2}}({M}_{z}\frac{{\rm{\partial }}{M}_{x}}{{\rm{\partial }}x}-{M}_{x}\frac{{\rm{\partial }}{M}_{z}}{{\rm{\partial }}x})$^19^, and substituting *M*_*x*_ and *M*_*z*_, we obtain ${\omega }_{{\rm{DM}}}=-\frac{{D}^{2}}{2A}$. As a result, the analytical expression of the DMI energy, which is ${E}_{{\rm{DMI}}}={\omega }_{{\rm{DM}}}\cdot V=-\frac{{D}^{2}V}{2A}$, gives the following results: *E*_DMI_ = −2.8 × 10^−17^ and −7.2 × 10^−17^ J for *D* = 2.5 and 4 mJ/m^2^, respectively. The very small discrepancy between both methods demonstrate the reliability of our results.

### Influence of the interfacial exchange constant

**Figure 3:**
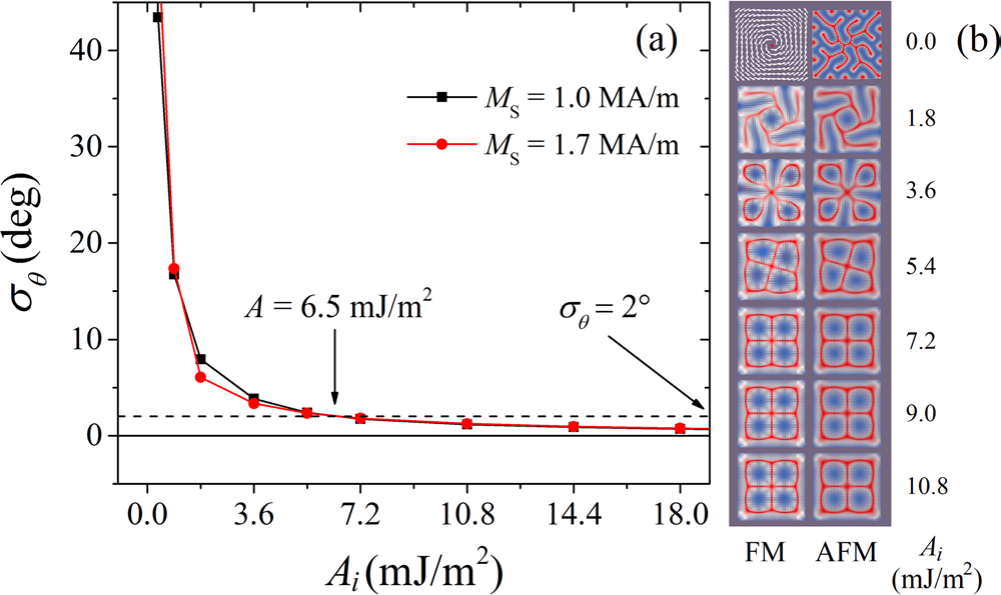
(**a**) Standard deviation curves of the angle difference between the magnetic moments in the FM and AFM interface layers, for different *A*_*i*_ and *M*_S_ values with *D* = 4 mJ/m^2^. A standard deviation of 2° leads to a critical *A*_*i*_ = 6.5 mJ/m^2^. (**b**) Magnetic moment distribution within the ferromagnetic and antiferromagnetic films as a function of the interfacial exchange constant *A*_*i*_ (in mJ/m^2^), for *D* = 4.0 mJ/m^2^ and *M*_S_ = 1.0 MA/m. The pattern taken for the AFM film is from the atomic layer adjacent to the FM film. Above *A*_*i*_ = 0 J/m^2^, the patterns of both films are almost identical, with only the magnitude being different (vectors in the AFM film are shorter), as their *M*_S_ are different. From *A*_*i*_ = 7.2 mJ/m^2^, the pattern, composed of four skyrmions, is almost completely stabilized.

It has been seen that neither the FM nor AFM film can generate skyrmions on its own. It will be interesting to see how the magnetic behavior of the system evolves if we introduce interfacial exchange between both films. The standard deviations of the angle difference between the magnetic moments in the FM and AFM interface layers, for different *A*_*i*_ and *M*_S_ values with *D* = 4 mJ/m^2^, are shown in Fig. 3(a). The FM and AFM films can be easily fully coupled with each other. The standard deviation drops rapidly to 2° at a weak interfacial exchange *A*_*i*_ = 6.5 mJ/m^2^ (equivalent to 3.6 pJ/m), which is much smaller than those in the films (*A*_FM_ = 14.5~25 pJ/m, *A*_AFM_ = 10 pJ/m). However, a partial coupling is sufficient to make the magnetic patterns similar to each other in both films. From our simulations, it appears that the distribution of magnetic moments in FM and AFM films are similar above *A*_*i*_ = 0.05, 0.2 and 0.5 mJ/m^2^ for *D* = 1, 2.5 and 4 mJ/m^2^, respectively. As an example, Fig. 3(b) demonstrates the magnetic patterns for different *A*_*i*_, with *D* = 4 mJ/m^2^ and *M*_S_ = 1.0 MA/m. We can see that the partially coupled magnetic patterns vary with the interfacial exchange (*A*_*i*_ = 1.8~5.4 mJ/m^2^). In contrast, the magnetic patterns are stabilized when the interfacial moments are fully coupled (*A*_*i*_ ≥ 7.2 mJ/m^2^) and transform from stripe-like to be flower-like.

**Figure 4:**
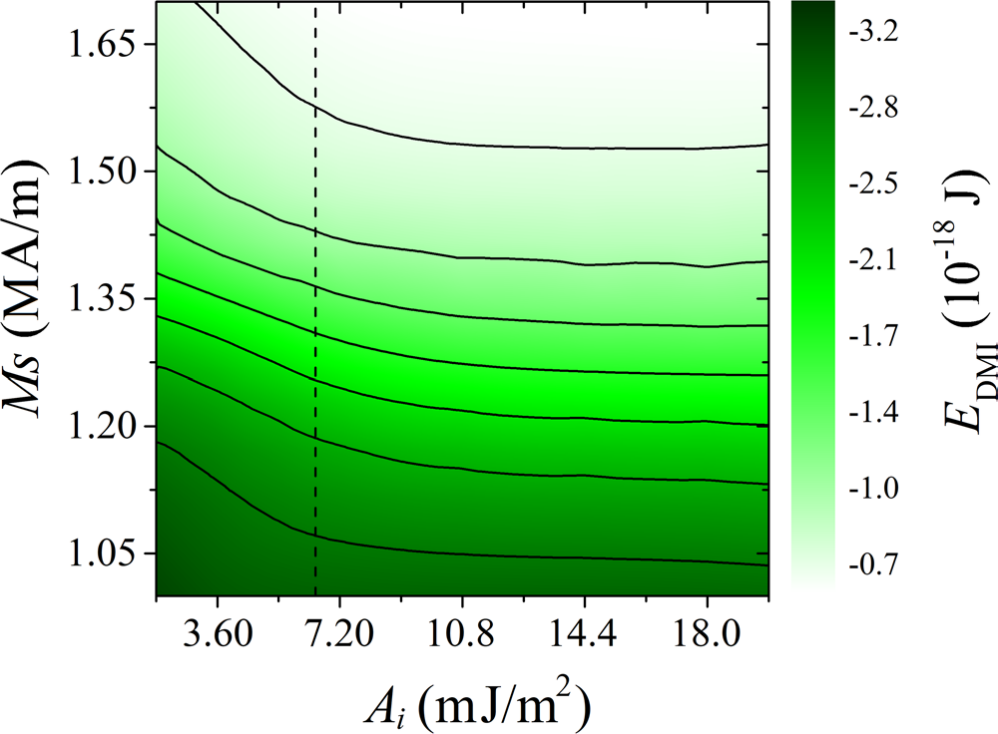
Contour graph of the DMI energy as functions of the saturation magnetization *M*_S_ and interfacial exchange constant *A*_*i*_, for *D* = 2.5 mJ/m^2^. No external field was applied. The dashed line represents the critical value above which the influence of *A*_*i*_ on the DMI energy is negligible.

To obtain a further understanding of the mechanism behind the evolution of the magnetic moment distribution, the total DMI energy in the AFM film was plotted as a function of both *A*_*i*_ and *M*_S_ (for *D* = 2.5 mJ/m^2^), as shown in Fig. 4. In this contour graph, the critical value *A*_*i*_ = 6.5 mJ/m^2^ shown in Fig. 3(a) can be seen more clearly. We want to emphasize that this is the maximum critical interface exchange for the whole range of investigated *D* values. Thus, above this value, the interfacial moments are always fully coupled with those on the other side, behaving like rigidly aligned with each other. Further increase of the interfacial exchange *A*_*i*_ has no significant influence on the DMI energy.

Below *A*_*i*_ = 6.5 mJ/m^2^, the DMI energy has a 10% to 20% discrepancy from *A*_*i*_ = 1.8 to 6.5 mJ/m^2^. In Fig. 4, the DMI energy gets higher as *A*_*i*_ increases towards the critical value for a given *M*_S_. A larger gradient of variation in DMI energy can be found for a higher *M*_S_. Indeed, as the coupling between the FM and AFM films becomes weaker, the magnetic alignment of the FM film (originating from the magnetostatic effect) has a lower influence on that of the AFM film, so that the DMI energy can reach lower values. It should be noted that if no other effects are present, the DMI energy itself favors stripe-like domains when its value is larger than a threshold^26^, as shown in Fig. 2 for *D* = 2.5 and 4 mJ/m^2^.

### Skyrmion nucleation under zero field

**Figure 5:**
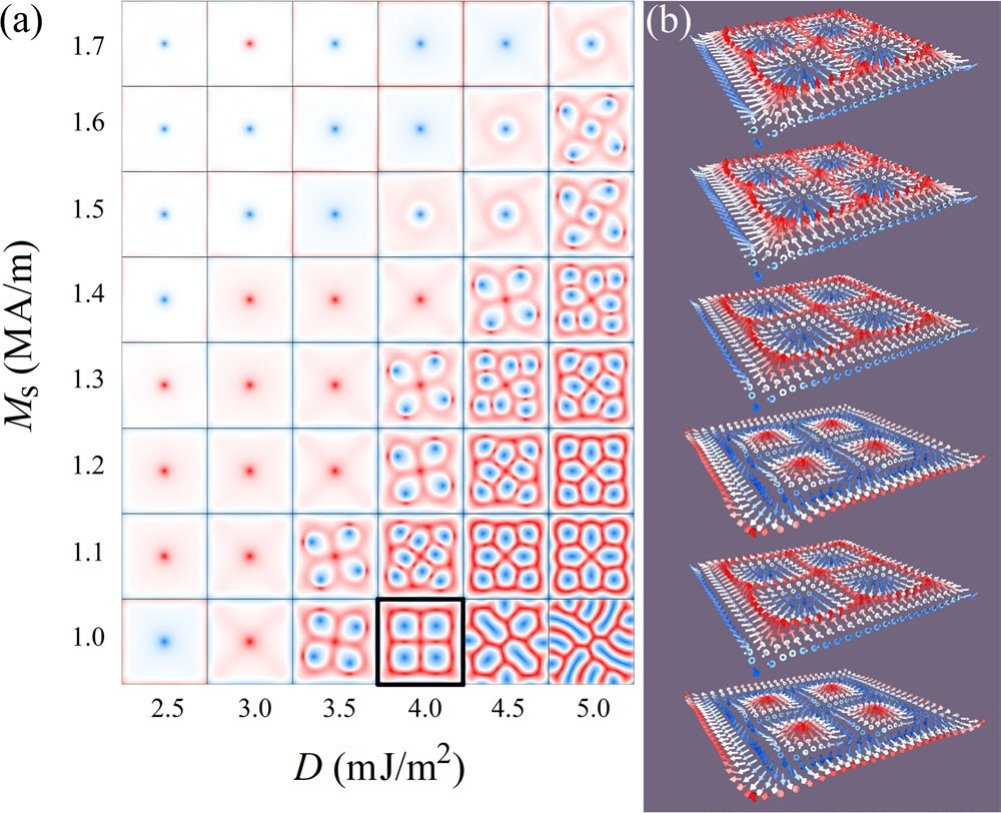
(**a**) Phase diagrams of the magnetic moment distribution as functions of the DMI constant *D* (in mJ/m^2^), and saturation magnetization *M*_S_ (in MA/m) of the ferromagnetic film, for *A*_*i*_ = 10.8 mJ/m^2^, under zero field. (**b**) Magnetic pattern distribution among the six atomic layers, for *A*_*i*_ = 10.8 mJ/m^2^, *D* = 4.0 mJ/m^2^ and *M*_S_ = 1.0 MA/m.

With a full coupling between the FM and AFM films, it can be expected that the magnetic behavior is only influenced by *D* and *M*_S_. Thus, a phase diagram of the magnetic patterns as functions of *D* and *M*_S_ is shown in Fig. 5(a), with the exchange interaction fixed at *A*_*i*_ = 10.8 mJ/m^2^ to obtain full coupling, so that the entire influence of *D* and *M*_S_ on the magnetic moment distribution can be investigated. In the phase diagram, as *M*_S_ goes lower and *D* goes higher, vortex states transit towards flower-like patterns. However, if *M*_S_ goes below 1.1 MA/m and *D* goes above 4.5 mJ/m^2^, they transform into stripe domains. Such a high DMI constant was taken into account in our simulations to have a broader picture of the skyrmion formation range. A typical stabilized flower-like pattern is clearly a cluster of Néel-type skyrmions, as shown in Fig. 5(b). This is also demonstrated by the calculated topological charge via $Q=1/4\pi \,{\iint }^{}[{\bf{m}}\cdot (\partial {\bf{m}}/\partial x\times \partial {\bf{m}}/\partial y)\,{\rm{d}}x{\rm{d}}y]$, with **m** = ***M***/*M*_S_ being the normalized magnetization. Each magnetic skyrmion in the pattern has an average numerical topological charge as high as 0.88, with an acceptable underestimate (compared to unity) coming from the free boundary condition^39^. Namely, the ideal topological charge of each magnetic skyrmion should be 1. This result is very interesting as here our model utilizes another mechanism, i.e. a weak exchange interaction between the FM and AFM films, so that neither a strong perpendicular anisotropy, nor a high exchange energy, nor an applied field are required to obtain skyrmions, while at least one of them is usually necessary^2,14,40,41^.

It is worth noting that the skyrmions in the clusters shown in the phase diagram are more or less distorted. This distortion is caused by the demagnetization energy which is high enough to prevent the vortex state from vanishing, and also by the DMI energy which favors the formation of stripe domains. The demagnetization energy is mainly influenced by the saturation magnetization and geometric confinement effect coming from the edges of the sample. Hence, a delicate combination of *M*_S_ and *D* is appreciated to obtain skyrmion clusters with minimal distortion, as distorted skyrmions have large size and are easy to be destroyed^42^. Such mechanism for the formation of elliptical skyrmions is different from that found in a strongly confined geometry^43^, or in samples with modified intrinsic magnetic interactions^42^.

### The energy competition at the origin of skyrmion formation

It has been found from the phase diagrams that the magnetic pattern tends to be a vortex for a large magnetization combined with a weak DMI, while it prefers a multi-domain pattern for the opposite. Note that the AFM film generates Néel-type spin spirals on its own due to the DMI, and the FM film forms magnetic vortex alone due to the reduction of demagnetization energy. A competition between them must be present if the interfacial exchange coupling is at its full extent. This is consistent with our findings presented in Fig. 5(a). At a high *M*_S_ and weak *D*, the requirement to reduce the demagnetization energy will dominate the magnetic distribution. Magnetic moments thus lie in the film plane to reduce the shape anisotropy energy. On the contrary, when *D* is very high and *M*_S_ is low, the pattern simply follows the magnetic behavior in the AFM film as DMI is very strong. Between these two extreme cases, the intermediate states will be featured by a compromise between the DMI in the AFM film and the demagnetization effect in the FM film. This is clearly shown by the evolution of the magnetic distribution in Fig. 5(a) (e.g., *D* = 4 mJ/m^2^) as the severely distorted skyrmion patterns are mixed states of the in-plane configurations and the Néel-type spirals.

### Influence of the external magnetic field

**Figure 6:**
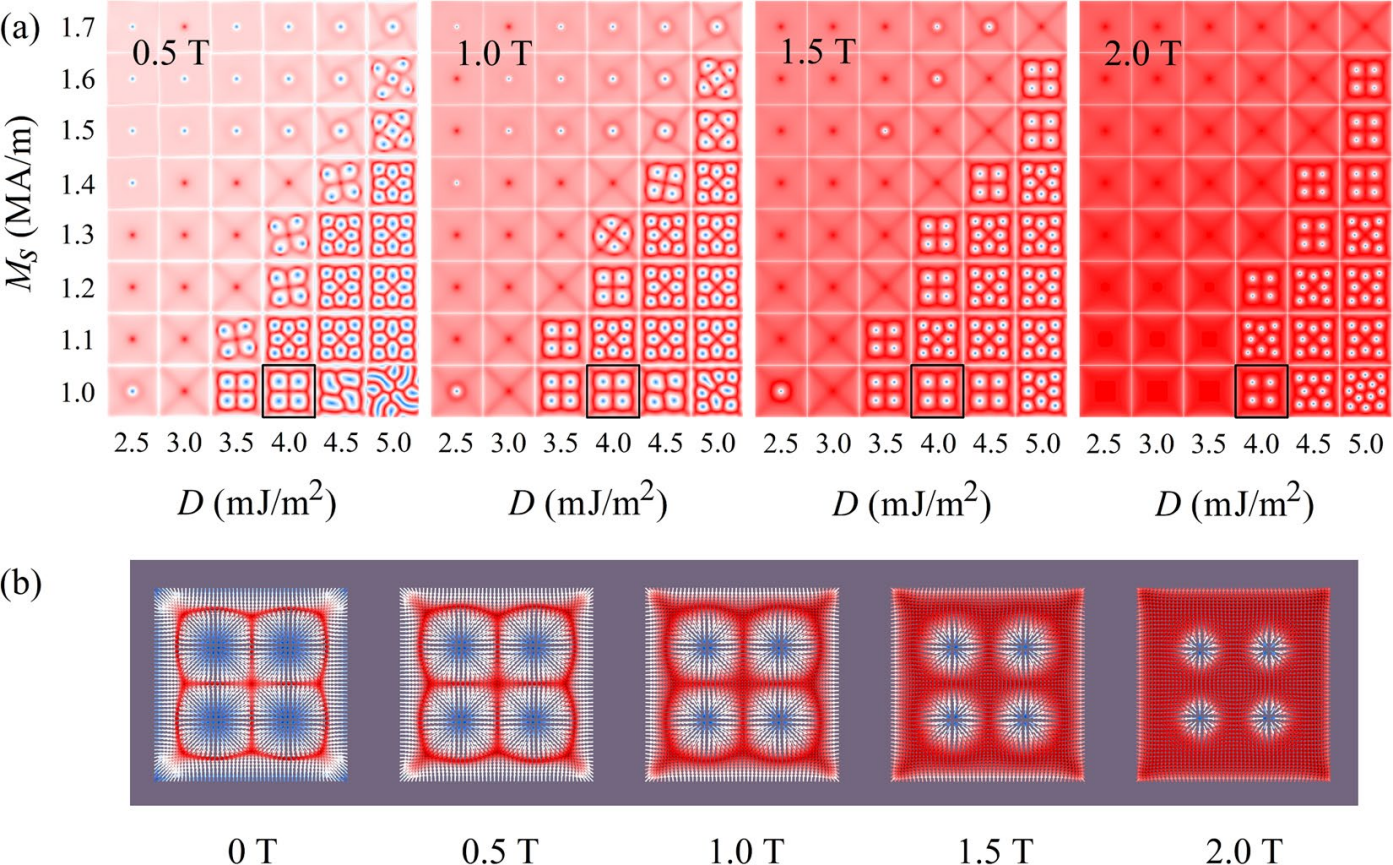
(**a**) Phase diagrams of the magnetic moment distribution as functions of the DMI constant *D* (in mJ/m^2^), saturation magnetization *M*_S_ (in MA/m) of the ferromagnetic film, and magnetic field *H* (in T), for *A*_*i*_ = 10.8 mJ/m^2^. (**b**) Evolution of the magnetic moment distribution as a function of the magnetic field, for *D* = 4 mJ/m^2^, *M*_S_ = 1.0 MA/m, and *A*_*i*_ = 10.8 mJ/m^2^. The corresponding patterns in the phase diagrams of (**a**) are outlined in black.

Although an external magnetic field is not necessary for the stabilization of skyrmions, it is still interesting to investigate its influence on their magnetic moment distribution. Figure 6(a) shows the phase diagrams for *H* = 0.5, 1.0, 1.5 and 2.0 T, respectively, as functions of *D* and *M*_S_. Such high *H* were taken into account to have a broader view of the external field influence. It can be found that the distortion and radius of skyrmions is reduced by the external magnetic field, as demonstrated in Fig. 6(b). Under high external magnetic field, some skyrmions disappear due to the polarization.

**Figure 7:**
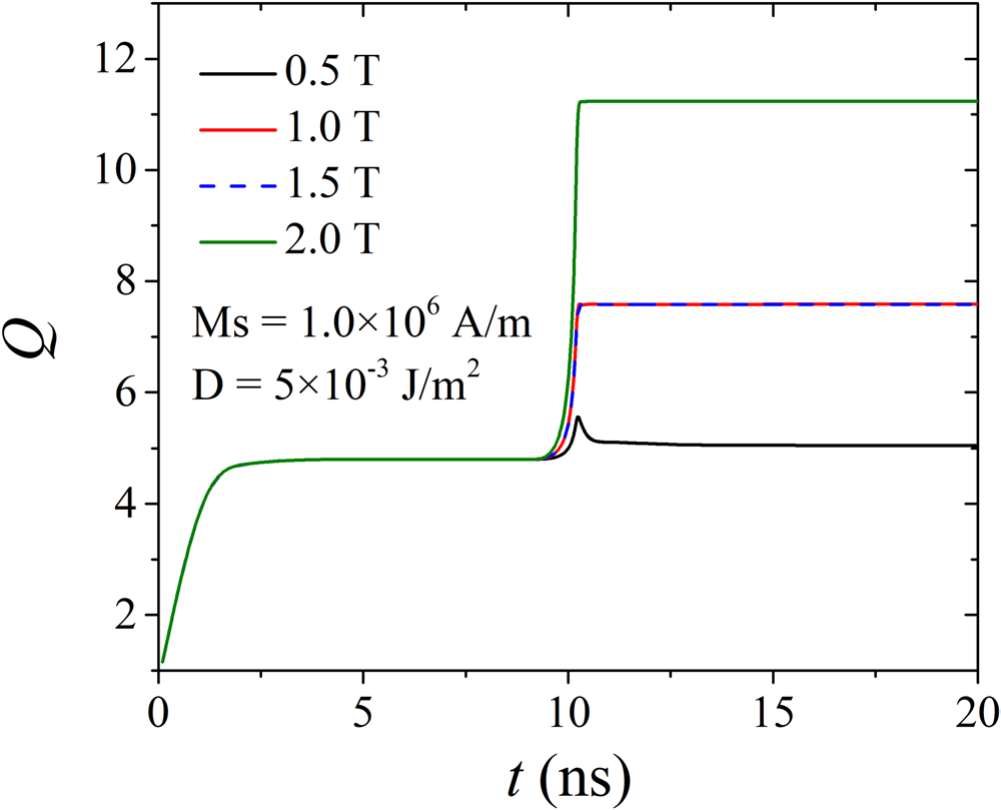
Influence of the external field on the topological charge *Q*, for *M*_S_ = 1.0 MA/m, *D* = 5.0 mJ/m^2^, and *A*_*i*_ = 10.8 mJ/m^2^. Four different external fields were applied, namely *H* = 0.5, 1.0, 1.5 and 2.0 T. For each curve, the system is first relaxing for 10 ns, then the external field is applied for 10 ns, making the simulation total running time 20 ns.

In the cases with a low *M*_S_ and a very high *D*, under zero field, only stripe domains are formed, whereas clusters of eight and twelve regular skyrmions occur under *H* = 1.5 and 2.0 T, respectively. It should be mentioned that skyrmions in most chiral magnets were derived from stripe domains by external field^2,14,40,41^. Here, we have shown that skyrmions can be induced in a non-chiral FM film with the help of an exchange-coupled chiral AFM film. A more interesting finding is that the number of skyrmions can be changed through the application of an external field. Taking the case with *M*_S_ = 1.0 MA/m and *D* = 5.0 mJ/m^2^ as an example, the number of skyrmions increases from eight for *H* = 1.0 and 1.5 T to twelve for *H* = 2.0 T. Note that initially there are twelve stripe domains in the pattern. Hence, under a lower field, some stripe domains merge to form skyrmions, while under a higher field, they independently shrink to be skyrmions. A possible reason is that a strong external field results in fast realignment of the magnetic moments to lower the Zeeman energy which does not allow equilibration. This change of number of skyrmions due to an external magnetic field is also reflected by the evolution of the numerically calculated topological charge, as given in Fig. 7. The stabilized *Q* is close to 8 for *H* = 1.0 and 1.5 T, while it is close to 12 for *H* = 2.0 T. With the highest total topological charge, *H* = 2.0 T with the parameters *D* = 5.0 mJ/m^2^ and *M*_S_ = 1.0 MA/m yields a promising inter-skyrmion distance of 45 nm, with a skyrmion diameter (circle of *m*_*z*_ = 0) as small as 14 nm and a density of 300 skyrmions per μm^2^.

From an application point of view, a high skyrmion density is strongly preferred. To increase the skyrmion density, one must reduce the inter-skyrmion distance, which depends on the skyrmion size and on the repulsion between skyrmions. Zhang X. *et al*. reported a 52 nm inter-skyrmion distance with a skyrmion diameter of 8 nm, and attributed the short distance to the small DMI helix length along with high perpendicular magnetic anisotropy^44^. Therefore, tuning the skyrmion distance requires a change of the materials properties. Interestingly, our results show that both the skyrmion size and the inter-skyrmion distance can be effectively tuned by the external field alone. For instance, the skyrmion diameter drops from 19 nm, associated with an inter-skyrmion distance of 57 nm, to 14 nm, associated with an inter-skyrmion distance of 45 nm, when the external field increases from 1.5 to 2.0 T for *D* = 5.0 mJ/m^2^ and *M*_S_ = 1.0 MA/m. This feature provides a way of on-fly manipulation of the skyrmion density.

### The linear equation binding skyrmion formation, *M*_S_ and DMI

**Figure 8:**
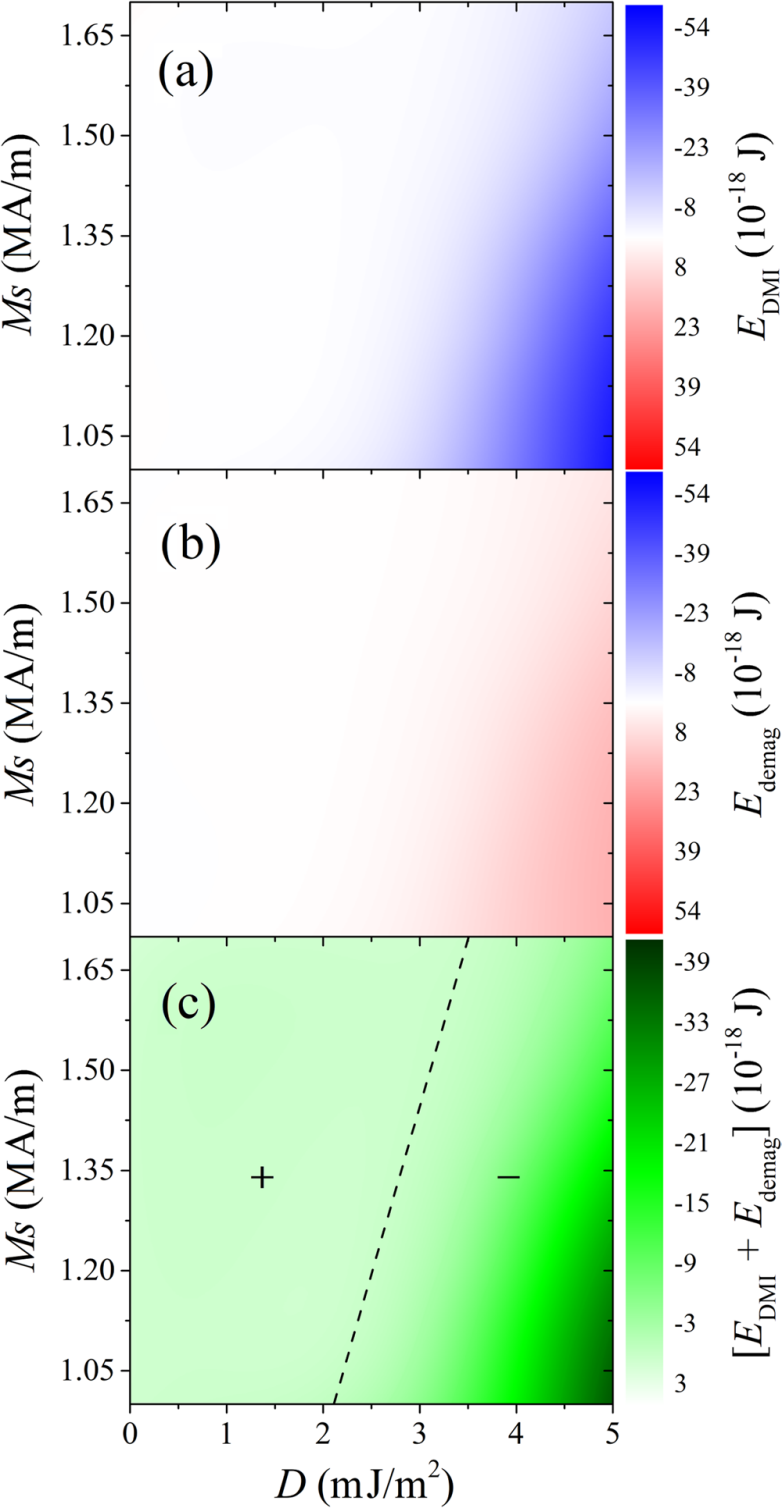
Contour graphs of (**a**) the DMI energy, (**b**) the demagnetization energy and (**c**) the sum of the DMI and demagnetization energies, as functions of the saturation magnetization *M*_S_ and DMI constant *D*. Results were calculated for *A*_*i*_ = 10.8 mJ/m^2^, under zero field.

In Fig. 8, we have shown the energy contours of DMI and demagnetization energies as functions of *D* and *M*_S_ (*H* = 0 T). The DMI energy decreases while the demagnetization energy increases with increasing *D* for a given *M*_S_ (Fig. 8(a,b)). Since DMI energy is always negative and demagnetization energy is always positive, they can fully cancel out at certain combinations of *D* and *M*_S_, as shown by the dashed line in Fig. 8(c). Hence, on the positive side of the line the magnetic pattern is dominated by the demagnetization effect, while on the other side it relies more on the DMI (exchange energy does not vary much). It is very interesting to see that this line lies close to the border between the skyrmion state and the nonchiral state in Fig. 5(a). Actually, the nonchiral state tranforms to skyrmions when the relation *E*_DMI_ + *E*_demag_ ≤ −7.6 × 10^−18^ J is satisfied. Assuming that the phase border can be treated as *M*_S_(*D*) = *a∙D* + *b*, we obtain the fitting parameters for different external fields as shown in Table 1. The fitting parameters are identical for *H* ≤ 1.5 T. The intercept (*b*) is very close to zero as there is no skyrmion for *M*_S_ = 0 A/m. However, when *H* reaches 2.0 T, the slope (*a*) increases from 0.32 to 0.4 due to the polarization of the skyrmion states with small *D* and low *M*_S_. The intercept also drops to be negative. These results provide a guidance to the future experimental design of the bilayer system with skyrmions.

**Table 1:** Linear fitting parameters *a* and *b* with their corresponding error margin for the *M*_S_(*D*) = *a*∙*D* + *b* equation, calculated for the phase border curves from the phase diagrams for *H* = 0.0, 0.5, 1.0, 1.5 and 2.0 T.

External field	*a*	*b*
*H* = 0.0 T	0.32 (±0.03)	−0.01 (±0.12)
*H* = 0.5 T		
*H* = 1.0 T		
*H* = 1.5 T		
*H* = 2.0 T	0.40 (±10^−16^)	−0.40 (±10^−15^)

## Conclusion

In summary, we have investigated the formation of skyrmions in a proposed FM/AFM system from which the DMI energy comes only from the AFM film. Our results show that skyrmions can be spontaneously generated without an external magnetic field, under a low interfacial exchange (no less than 6.5 mJ/m^2^) between the two films. The skyrmions exist in a wide range of DMI in the AFM film and saturation magnetization in the FM film, although they could be distorted. Applying an out-of-plane external magnetic field can reduce the distortion of skyrmions by shrinking their size. A higher external magnetic field generally results in more skyrmions, until a critical value where the external magnetic field annihilates the existing skyrmions. Overall, it is discovered that through an AFM film with large bulk DMI, an FM film with low *M*_S_ and a certain out-of-plane external magnetic field, the mean inter-skyrmion distance can be as small as 45 nm, which is a very striking result. Our simulation results demonstrate that the number of skyrmions formed in our model system can be tuned to reach a high density. The next step should be to experimentally demonstrate the feasibility of such a model, as well as to design a racetrack utilizing such an FM/AFM system.

### Methods

All the micromagnetic simulations are performed using the 1.2 beta 0 release of the Object-Oriented MicroMagnetic Framework (OOMMF) software^45^, including the extension module to model DMI^26^. This Néel DMI module was used in these simulations to model the Néel spiral magnetization discovered in AFM materials with bulk DMI^34^. Without spin-polarized current, the 3D magnetization dynamics in the FM film is governed by the standard Landau-Lifshitz-Gilbert (LLG) equation^45–47^

$$\frac{d{\boldsymbol{M}}}{dt}=-\,{\gamma }_{0}\,{\boldsymbol{M}}\times {{\boldsymbol{H}}}_{eff}+\frac{\alpha }{{M}_{{\rm{S}}}}({\boldsymbol{M}}\times \frac{d{\boldsymbol{M}}}{dt}),$$

where ***M*** is the magnetization, *M*_S_ = |***M***| is the saturation magnetization, *t* is the time, *γ*_0_ is the gyromagnetic ratio with absolute value, and *α* is the Gilbert damping coefficient. ***H***_*eff*_ is the effective field, which reads^48^:

$${{\boldsymbol{H}}}_{eff}=-\,{\mu }_{0}^{-1}\frac{{\rm{\partial }}}{{\rm{\partial }}{\boldsymbol{M}}}\{A{[{\rm{\nabla }}(\frac{{\boldsymbol{M}}}{{M}_{{\rm{S}}}})]}^{2}-K\frac{{({\boldsymbol{M}}\cdot {\boldsymbol{n}})}^{2}}{{M}_{{\rm{S}}}^{2}}-\frac{1}{2}{\mu }_{0}{{\boldsymbol{H}}}_{{\boldsymbol{d}}}({\boldsymbol{M}})\cdot {M}_{{\rm{S}}}-{\mu }_{0}{\boldsymbol{M}}\cdot {\boldsymbol{H}}+{\omega }_{{\rm{D}}{\rm{M}}}\cdot V\}$$

where *A* and *K* are the exchange and anisotropy energy constants, respectively. The five terms in the braces are the Heisenberg exchange energy, the magnetic anisotropy energy, the demagnetization energy, the Zeeman energy and the DMI energy. The DMI energy density (in J/m^3^) in a continuous magnetization model is expressed as^19^:

$${\omega }_{{\rm{D}}{\rm{M}}}=\frac{D}{{M}_{{\rm{S}}}^{2}}({M}_{z}\frac{{\rm{\partial }}{M}_{x}}{{\rm{\partial }}x}-{M}_{x}\frac{{\rm{\partial }}{M}_{z}}{{\rm{\partial }}x}+{M}_{z}\frac{{\rm{\partial }}{M}_{y}}{{\rm{\partial }}y}-{M}_{y}\frac{{\rm{\partial }}{M}_{z}}{{\rm{\partial }}y})$$

where *M*_*x*_, *M*_*y*_ and *M*_*z*_ are the components of the magnetization *M* and *D* is the continuous effective DMI constant.

The width of the square nanostructure is set as 200 nm, and the thickness as 3.36 nm, corresponding to six 0.56-nm-thick layers, with two and four layers for the FM and AFM films, respectively. All models are discretized into 2 × 2 × 0.56 nm^3^ cells. The simulation parameters are as follows:

(1) The FM film parameters^49^ are: saturation magnetization *M*_S_ = 1.0~1.7 MA/m, magnetic anisotropy constant *K* = 0.046 mJ/m^3^ on the *z*-axis, and exchange constant *A*_FM_ set proportional to the *M*_S_ value, from *A* = 14.5 pJ/m for *M*_S_ = 1.0 MA/m to *A* = 25 pJ/m for *M*_S_ = 1.7 MA/m. The *A* value has an increment of 1.5 pJ/m between these two extreme values. The initial state for the FM film is a ferromagnetic state on the *z*-axis.

(2) An AFM material is composed of alternately stacking layers with negative interlayer exchange coupling^50^, which explains our simulation model as shown in Fig. 1. Our model is valid for the *G*-Type AFM materials proposed in the introduction as they also have stacked FM layers in the (111) plane. The negative interlayer exchange constant was set as −10 pJ/m, and the intralayer exchange constant was set as 10 pJ/m. This effectively corresponds to a high Néel temperature of 640 K, which is close to some AFM oxides with DMI^34,38^. Each layer has a saturation magnetization *M*_S_(AFM) which was calculated following its definition, namely, it is a volume density of magnetic moments. Dividing the sum of the magnetic moments by the lattice volume led to *M*_S_(AFM) = 0.54 MA/m^51^. Our FM film will at least have a saturation magnetization *M*_S_ = 1.0 MA/m. The saturation magnetization of the AFM film should indeed be lower as there are nonmagnetic elements in AFM materials. Lastly, DMI constant *D* = 0~5 mJ/m^2^. Such high DMI constants were taken in order to have enough data to get an equation of the skyrmion creation trend. The initial state of the AFM film is composed of an alternation of *−z* and +*z* ferromagnetic states, as shown in Fig. 1.

(3) Other parameters for both films: Gilbert damping constant *α* = 0.3, external field *H* = 0.0~2.0 T (same as for the DMI constant, high *H* were taken to have a better overview of the skyrmion creation trend), and interfacial exchange between the FM and AFM films *A*_*i*_ = 0~21.6 mJ/m^2^^49^.
